# The effects of hedgehog ligand neutralising antibody 5E1 in a mouse model of endometriosis

**DOI:** 10.1186/s13104-020-05299-5

**Published:** 2020-09-25

**Authors:** F. L. Cousins, J. K. Farley, R. Kerrigan, S. Mukherjee, S. Darzi, C. E. Gargett, J. A. Deane

**Affiliations:** 1 The Ritchie Centre, Hudson Institute of Medical Research, 27-31 Wright Street, Clayton, VIC Australia; 2 Department of Obstetrics and Gynaecology, School of Clinical Sciences At Monash Health, Monash University Faculty of Medicine, 246 Clayton Road, Clayton, 3168 Australia

**Keywords:** Endometrium, Endometriosis, Hedgehog signalling, Mouse model

## Abstract

**Objective:**

Endometriosis is a common and painful condition characterised by the formation of endometrial lesions within the peritoneal cavity. Previous studies have suggested a role for hedgehog signalling in the pathogenesis of endometriosis. We investigated the role of hedgehog signalling in the establishment of endometriosis lesions using 5E1, a hedgehog ligand neutralising antibody, and a mouse model of endometriosis. To mimic the initiation of endometriosis by retrograde menstruation, which is believed to occur in humans, donor mice underwent an artificial menstruation protocol. Fragments of menstrual endometrium were injected into the peritoneal cavity of estrogen primed recipients. Recipients received twice weekly injections of 5E1 or an isotype matched control antibody for three weeks. Lesions were collected and analysed for markers of epithelium, proliferation and apoptosis by immunofluorescence microscopy.

**Results:**

Treatment with 5E1 reduced the number of lesions found on the mesentery. No significant changes were found in the size of lesions, abundance of endometrial epithelial cells, proliferation or apoptosis.

## Introduction

Endometriosis is a complex disorder of unknown aetiology, defined by the growth of endometrial fragments outside the uterine cavity. Endometriosis is thought to occur via the retrograde menstruation of endometrial fragments into the peritoneal cavity which then persist and form lesions on intra-peritoneal organs [1]. Retrograde menstruation occurs in 90% of menstruating females, yet only 10% go on to develop endometriosis, indicating that additional factors are involved [2]. Published data highlights that eutopic endometrial stromal cells from women with endometriosis exhibit increased adherence and proliferation in vitro [3, 4]. It is likely that cell signalling and cell growth pathways of eutopic endometrial cells are altered in women with endometriosis. Current non-surgical treatments for endometriosis are largely oral contraceptives, to limit retrograde menstruation and growth of established lesions, or analgesics for pain. Non-hormonal therapeutics are desperately needed and targeting cell signalling pathways may reveal new avenues for drug design.

Hedgehog signalling is a developmental pathway that is activated in some endometrial cancers [5] and the endometrium of women with endometriosis [6]. Sonic hedgehog (*SHH*) and its downstream signalling transcription factor *GLI1* are upregulated in the eutopic endometrium of women with endometriosis in comparison to healthy controls [6]. Hedgehog signalling genes *Shh, Gli1, Stil1* and *Jag2* are also upregulated in the ectopic lesions of a mouse model of endometriosis featuring enhanced lesion formation [7]. Hedgehog signalling regulates cell proliferation, differentiation and stromal fibroblast maintenance in the endometrium via the FAK/ERK1/2 and PI3K/Akt signalling pathways [8]. Both of these pathways have been implicated in the pathogenesis of endometriosis [9], with prolonged phosphorylation of ERK in endometrial epithelial [10] and stromal cells [11] and endometriomas [12] and increased phosphorylated Akt in ovarian endometriomas [13]. HOXA10 is a developmental transcription factor that has been linked to endometriosis, and, like hedgehog signalling, is involved in progesterone induced endometrial differentiation [14]. This evidence suggests that hedgehog signalling contributes to the pathogenesis of endometriosis. Thus, hedgehog signalling may have value as a diagnostic marker for endometriosis and as a therapeutic target.

Highly specific antibodies are increasingly being used in therapeutic applications [15]. The 5E1 monoclonal antibody recognises mammalian hedgehog ligands and prevents hedgehog signal transduction via the Patched receptor. 5E1 is commonly used as a tool to block hedgehog signalling in mouse-based in vivo experiments [16, 17]. In the current study we investigated the potential of hedgehog blocking antibodies as a therapy for endometriosis by using 5E1 in a mouse model that closely mimics the human condition.

## Main Text

### Materials and methods

#### Animal ethics

All animals were held in the Monash Medical Centre Animal Facility and housed on a 12-h light/day cycle with access to normal chow and water *ab libitum*. Approval for all procedures described was obtained from Monash Medical Centre Animal Ethics Committee A.

#### Mouse model of endometriosis

C57BL/6J background mice 8–12 weeks age underwent the mouse model of endometriosis as published previously [18]. Briefly, donor mice were ovariectomised, then given sub-cutaneous injections of β-estradiol (1 µg/ml) on days 7–9. A progesterone secreting pellet was placed sub-cutaneously from days 13–19 (500 ng/day) in combination with β-estradiol sub-cutaneous injections from days 13–15 (50 ng/ml). On day 15 the endometrium was artificial decidualised by injecting 20 µl of oil into the uterine lumen. Progesterone support was removed on day 19 and 4 h later the menstrual-like endometrium was removed from the outer myometrium, minced into fragments approximately 1 mm^3^ and injected into ovariectomised estrogen primed (β-estradiol secreting pellet, 100 ng/day days 7–19) recipients (approximately 20 fragments, 200 mg of tissue in 200 µl of PBS). Lesions were allowed to develop over 21 days (days 19–40) before collection on day 40. During this 21-day period mice were randomly allocated to one of two groups and treated twice weekly with either 250 µg of anti-SHH antibody 5E1 (n = 15, DSHB) or an isotype matched control antibody (n = 18, BioXCell, MOPC-21 IgG1) in 200 µl of sterile PBS. The 5E1 dose administered was based on previous reports that used this antibody to block hedgehog signalling in mouse in vivo tumour models [19].

#### Lesion collection and fixation

Recipient mice were euthanised using rising concentrations of carbon dioxide and cervical dislocation. Upon dissection all body cavities were photographed, the number of lesions found recorded and their size measured. Lesions were carefully removed from peritoneal organs and immersed in 4% w/v paraformaldehyde in PBS overnight at 4 °C and cryoprotected in 30% w/v sucrose in PBS overnight at 4 °C. Tissues were frozen in optimal cutting temperature medium and cryo-sectioned at 8 µm thick.

#### Immunofluorescence analysis

Unless otherwise stated all sections underwent the following staining protocol. Sections were permeabilised in 0.2% Triton X-100 in PBS for 15 min, blocked in DAKO blocking solution for 1 h and then stained for hedgehog activation, (GLI1 Rabbit monoclonal Thermo MA5-32553 10 µg/ml), epithelial (EpCAM-PE rat anti mouse eBioscience 12-5791-81 2 µg/ml), proliferation (Ki67-EF660 rat anti mouse eBioscience 50-5698-80 2 µg/ml) and apoptosis (Caspase 3 Rabbit polyclonal R&D AF835 5 µg/ml) markers for 1 h at room temperature in 1% bovine serum albumin in PBS. For unconjugated primary antibodies, sections were incubated with secondary antibodies for 1 h at room temperature (Donkey anti rabbit Alexafluor 568 LifeTech A10042 4 µg/ml). Nuclei were counterstained with 5 µg/ml Hoescht 33258 in PBS for 3 min. Images were captured on an Olympus FV1200 confocal microscope using a 20 × objective lens and adjusted for brightness and contrast in a linear manner using FIJI software [20].

#### Analysis of lesions

##### Histological analysis

Haematoxylin and eosin staining was performed as per standard protocols on the 15th slide of each lesion (2 sections/slide × 8 µm × 15 slides = 240 µm deep). Lesion cross-sectional area was measured using FIIJ, using the trace outline and measure area tools.

Prior to the analysis of epithelial, proliferation and apoptosis markers, slides were blinded to reduce any bias during analysis. Markers were counted manually in FIJI. Total cell nuclei were calculated by counting the number of nuclei using the “threshold”, “watershed” and “analyse particles” functions. A minimum of 6 lesions were analysed per treatment group. A minimum of 3 fields of view per section were imaged and the average percentage of positive cell types in the total cell population was calculated for each lesion. Results were then unblinded and each lesion was then plotted as a single data point.

#### Statistical analysis

Group sizes were determined using G*Power software. For lesion number, we assumed an observed effect size of 0.5, alpha of 0.05 and Power of 0.8, the total sample size required was 26. For changes in lesion characteristics we assumed an effect size of 0.65, alpha of 0.05 and Power of 0.8, the total sample size required was 13.

All statistical analyses were performed in Graphpad Prism 8.0. Raw data was subjected to D’Agostino-Pearson normality testing prior to statistical analyses. Non-parametric analysis was performed when one of the sets of data failed to pass normality testing. All lesion analyses were subjected to unpaired, two-tailed Mann–Whitney testing. Significance was accepted where P ≤ 0.05 and data represented graphically as individual data points and median.

### Results and discussion

#### The location and incidence of lesions

**Fig. 1:**
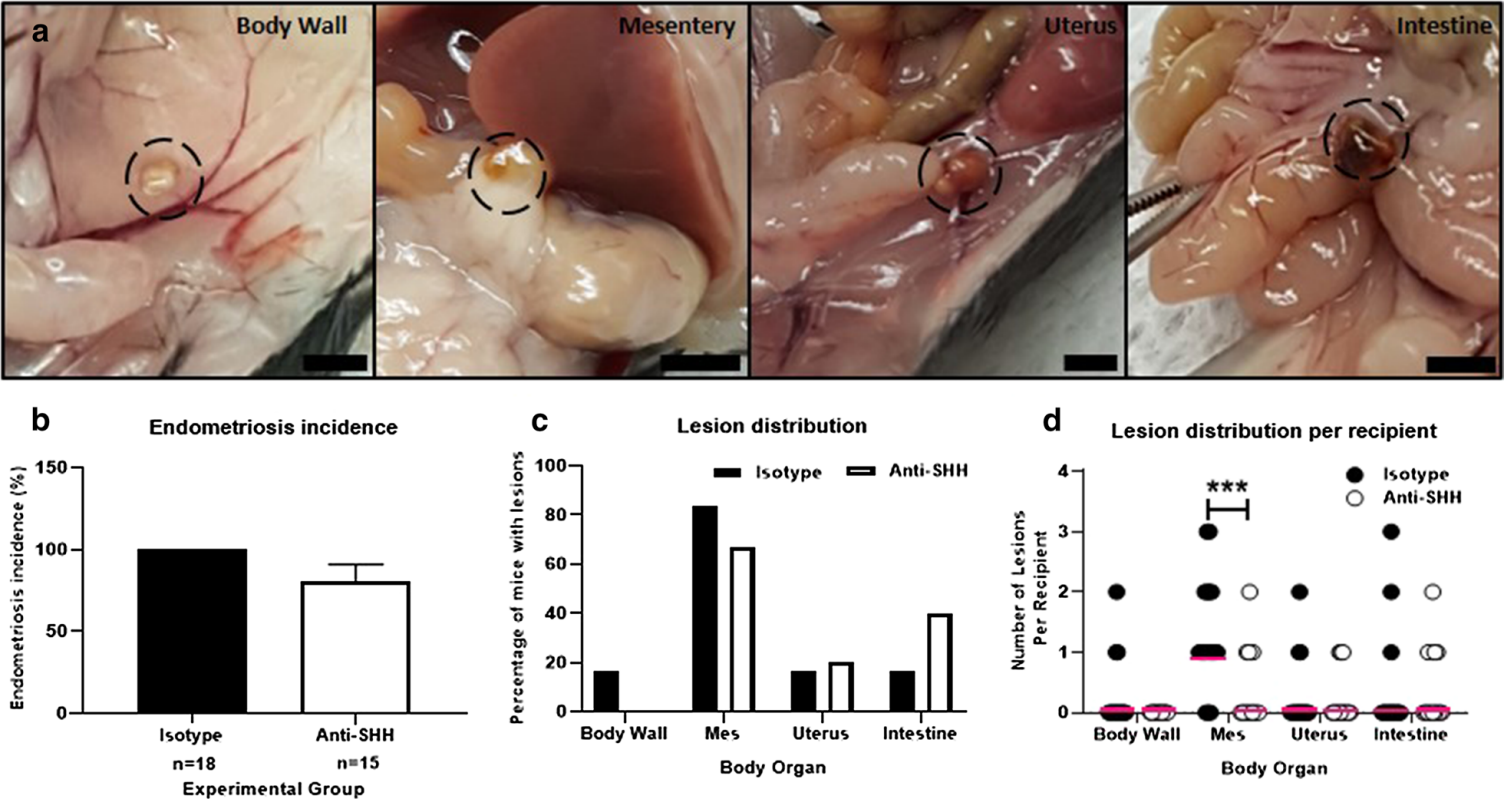
Endometriosis incidence and lesion location following treatment with anti-SHH antibody. **a** Lesions (dashed circles) were identified on different organs within the body cavity, including the body wall, mesentery, uterus and intestine. Scale bars 3 mm. **b** Endometriosis incidence was calculated as the percentage of mice with detectable lesions at three weeks, isotype control n = 18, anti-SHH n = 15. Data displayed as mean ± SEM. **c** The percentage of mice with lesions at different sites. **d** The number of lesions/recipient at each site. Isotype v anti-SHH for each site was tested using an unpaired, two-tailed Mann–Whitney test. ***p < 0.001. Pink line denotes median

Lesions were found at various locations in the isotype control and anti-SHH treated group (Fig. 1a). The occurrence of lesions, indicative of endometriosis, was 100% in the isotype control treated group and 80% in anti-SHH treated group (p = 0.08) (Fig. 1b). In line with other reports using this model, irrespective of treatment, most lesions were detected on the mesentery (83% in the isotype control group, 67% in the 5E1 group) [18]. Lesions were also found on the body wall, on the external surface of the uterus and on the digestive tract (includes stomach, small intestine, large intestine) (Fig. 1c). There was a significant decrease in the number of lesions per recipient detected on the mesentery after anti-SHH treatment compared to isotype control (p < 0.01) (Fig. 1d). This decrease was not observed for other locations but these lesions were infrequent and the dataset was small (Fig. 1d). Given the decrease in the number of mesenteric lesions we assessed their lesion area independent of other lesions but found that this sub-group did not display any difference in area (p = 0.0932, data not shown).

#### Gli1 expression as an indicator of hedgehog activation

**Fig. 2:**
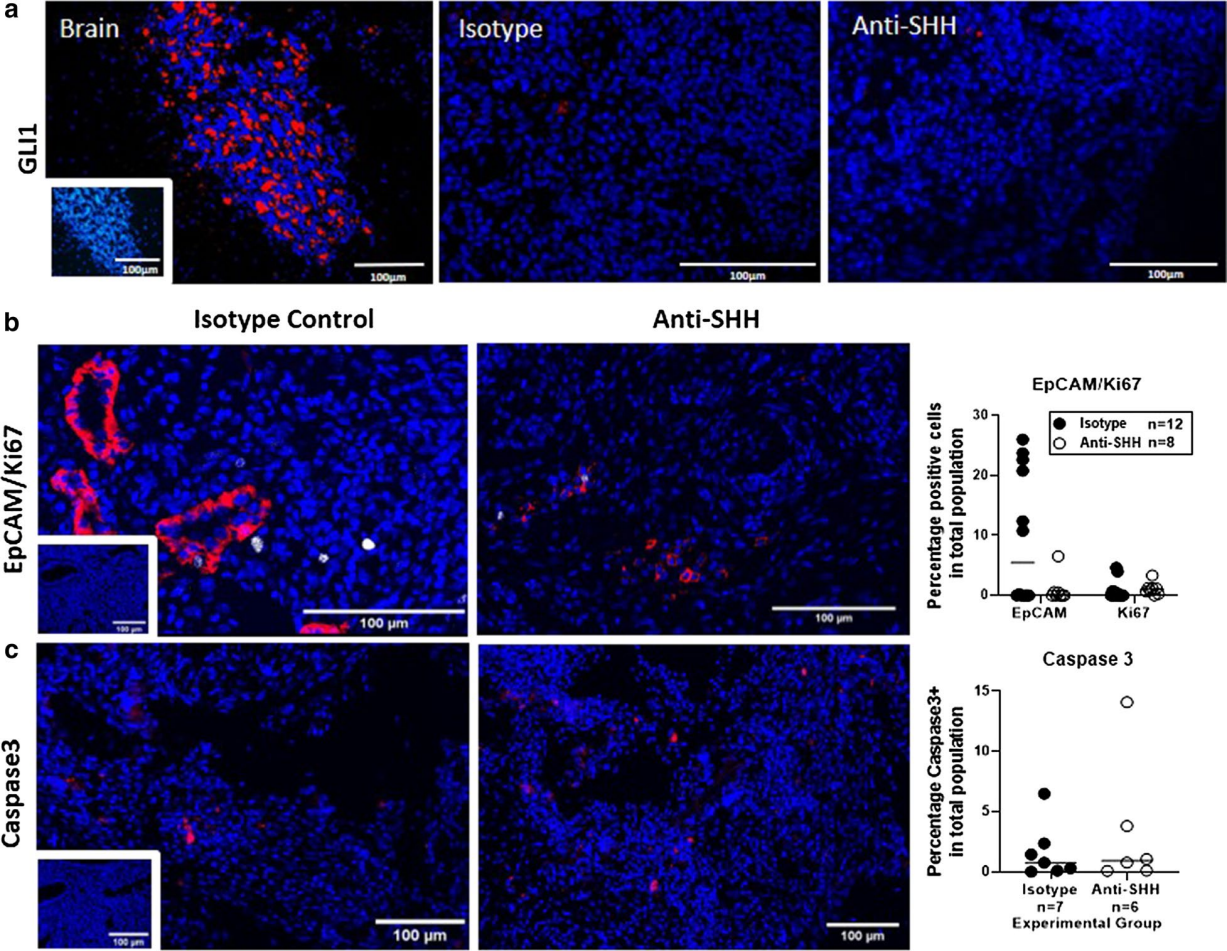
Expression of SHH pathway and cell identity markers did not change with anti-SHH treatment. **a** Gli1 expression (red) was detected in the mouse brain but was barely detected in lesions from either group. Inset rabbit IgG1 isotype control. **b** Dual immunofluorescence for EpCAM (red) and Ki67 (white), insert isotype control. **c** Immunofluorescence for Caspase 3 (red), insert isotype control. B + C quantification; EpCAM, Ki67 and Caspase 3 expressed as a percentage of total cell population, isotype control (black circles) and anti-Shh (white circles). Data analysed by an unpaired, two-tailed Mann–Whitney test

Gli1 was examined by immunostaining to detect hedgehog activation in collected lesions. Gli1 was readily detected in mouse brain, a known site of hedgehog signalling [21]. Very weak to no signal was detected in isotype and anti-SHH lesions from the endometriosis model (Fig. 2a, representative of 3 animals). This suggests that endometriotic lesions do not have the high levels of hedgehog activation found in some tumours [5, 22–24]. GLI1 expression is increased in the endometrium of women with endometriosis in comparison to healthy controls [6], and in ovarian endometriomas [25] but expression in human lesions at other sites is yet to be assessed. Our mouse model does not address ovarian endometriomas due to recipients being ovariectomised, therefore we cannot comment on whether the ovarian location would result in differing GLI1 expression. Furthermore, the human study used a polyclonal antibody, whereas the antibody used in our mouse studies was a monoclonal (clone JF09-08) which may account for discrepancy in results.

#### Lesion characteristics

Lesion establishment and survival relies on cross-talk between epithelial cells and the surrounding stroma [26]. Lesions were investigated for expression of the epithelial cell marker EpCAM, proliferation marker Ki67 and apoptosis marker Caspase 3 (Fig. 2b and c). Variable expression of EpCAM was identified in the isotype control group. Fifty-eight percent of isotype lesions (7 of 12) had detectable EpCAM expression ranging from 0.17–26% positive cells in the total population. In the anti-SHH group, only 37.5% of lesions had detectable expression of EpCAM (3 of 8 lesions), only 1 lesion had a detectable level over 1% positive in the total cell population. Despite no significant decrease in EpCAM expression, the observed downward trend is in line with another report where a hedgehog inhibitor decreased endometrial epithelial cell proliferation in vitro [27].

No significant differences were identified between isotype control and anti-SHH groups for Ki67 and Caspase 3 (Fig. 2b and c), which suggests 5E1 has no direct effect on overall proliferation or apoptosis of endometriotic lesions.

### Conclusions

Differences in hedgehog pathway activation have been detected in women with endometriosis and in mouse models of endometriosis [6, 7, 28]. This study aimed to see whether the hedgehog neutralising antibody 5E1 would prevent lesion establishment and progression in a mouse model of endometriosis. Significantly fewer lesions were found on the mesentery of animals treated twice weekly with 5E1. However, no significant differences were observed in the size and morphology of the lesions that formed. These data suggest that hedgehog signalling is not integral to lesion progression, but may play a role in the survival of menstrual fragments in the peritoneal cavity and lesion initiation.

## Limitations

No effect of anti-SHH treatment was observed at other sites within the peritoneal cavity, however the number of lesions obtained from these sites was small (n = 4–6 lesions) meaning it is not possible to draw clear conclusions. It is difficult to determine the mechanism by which mesenteric lesions are reduced from the data obtained.

## Data Availability

All relevant data generated in this study is presented in the manuscript.

## Abbreviations

5E1: Sonic hedgehog neutralising monoclonal antibody, Clone 5E1
EpCAM: Epithelial cell adhesion molecule
SHH: Sonic hedgehog
